# Interhemispheric imbalance and bradykinesia features in Parkinson’s disease

**DOI:** 10.1093/braincomms/fcae020

**Published:** 2024-01-29

**Authors:** Giulia Paparella, Martina De Riggi, Antonio Cannavacciuolo, Davide Costa, Daniele Birreci, Massimiliano Passaretti, Luca Angelini, Donato Colella, Andrea Guerra, Alfredo Berardelli, Matteo Bologna

**Affiliations:** IRCCS Neuromed, Pozzilli, IS 86077, Italy; Department of Human Neurosciences, Sapienza, University of Rome, Rome 00185, Italy; Department of Human Neurosciences, Sapienza, University of Rome, Rome 00185, Italy; IRCCS Neuromed, Pozzilli, IS 86077, Italy; Department of Human Neurosciences, Sapienza, University of Rome, Rome 00185, Italy; Department of Human Neurosciences, Sapienza, University of Rome, Rome 00185, Italy; Department of Human Neurosciences, Sapienza, University of Rome, Rome 00185, Italy; IRCCS Neuromed, Pozzilli, IS 86077, Italy; Department of Human Neurosciences, Sapienza, University of Rome, Rome 00185, Italy; Parkinson and Movement Disorders Unit, Study Center for Neurodegeneration (CESNE), Department of Neuroscience, University of Padua, Padua 35121, Italy; Padova Neuroscience Center (PNC), University of Padua, Padua 35131, Italy; IRCCS Neuromed, Pozzilli, IS 86077, Italy; Department of Human Neurosciences, Sapienza, University of Rome, Rome 00185, Italy; IRCCS Neuromed, Pozzilli, IS 86077, Italy; Department of Human Neurosciences, Sapienza, University of Rome, Rome 00185, Italy

**Keywords:** Parkinson’s disease, interhemispheric inhibition, motor cortex, bradykinesia, kinematic analysis

## Abstract

In patients with Parkinson’s disease, the connectivity between the two primary motor cortices may be altered. However, the correlation between asymmetries of abnormal interhemispheric connections and bradykinesia features has not been investigated. Furthermore, the potential effects of dopaminergic medications on this issue remain largely unclear. The aim of the present study is to investigate the interhemispheric connections in Parkinson’s disease by transcranial magnetic stimulation and explore the potential relationship between interhemispheric inhibition and bradykinesia feature asymmetry in patients. Additionally, we examined the impact of dopaminergic therapy on neurophysiological and motor characteristics. Short- and long-latency interhemispheric inhibition was measured in 18 Parkinson’s disease patients and 18 healthy controls, bilaterally. We also assessed the corticospinal and intracortical excitability of both primary motor cortices. We conducted an objective analysis of finger-tapping from both hands. Correlation analyses were performed to explore potential relationships among clinical, transcranial magnetic stimulation and kinematic data in patients. We found that short- and long-latency interhemispheric inhibition was reduced (less inhibition) from both hemispheres in patients than controls. Compared to controls, finger-tapping movements in patients were slower, more irregular, of smaller amplitudes and characterized by a progressive amplitude reduction during movement repetition (sequence effect). Among Parkinson’s disease patients, the degree of short-latency interhemispheric inhibition imbalance towards the less affected primary motor cortex correlated with the global clinical motor scores, as well as with the sequence effect on the most affected hand. The greater the interhemispheric inhibition imbalance towards the less affected hemisphere (i.e. less inhibition from the less to the most affected primary motor cortex than that measured from the most to the less affected primary motor cortex), the more severe the bradykinesia in patients. In conclusion, the inhibitory connections between the two primary motor cortices in Parkinson’s disease are reduced. The interhemispheric disinhibition of the primary motor cortex may have a role in the pathophysiology of specific bradykinesia features in patients, i.e. the sequence effect.

## Teaser

Paparella *et al*. observed an interhemispheric inhibition reduction among Parkinson’s disease individuals. This decrease in inhibitory connections between motor cortices correlated with clinical scores and the sequence effect on the more affected side. Hence, diminished interhemispheric inhibition appears to contribute to the pathophysiology of some bradykinesia features in Parkinson’s disease.

## Graphical Abstract

**Figure:**
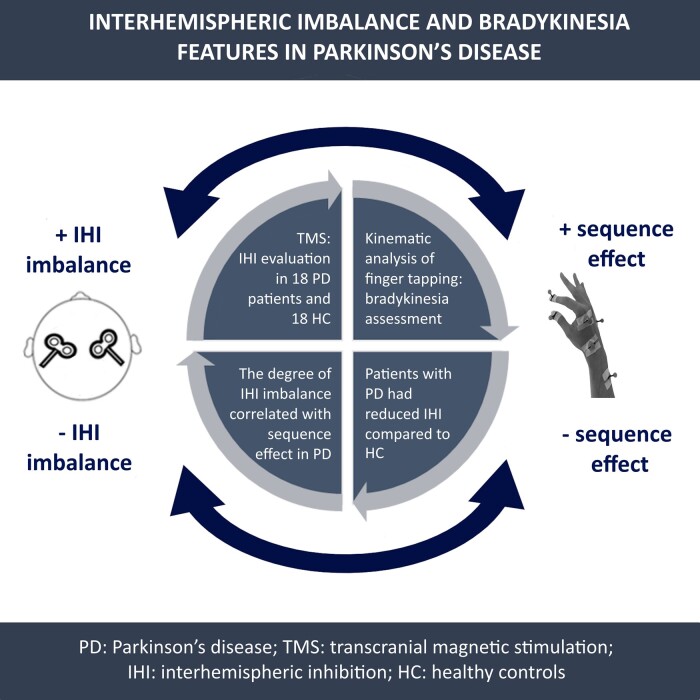

## Introduction

Bradykinesia, a prominent symptom of Parkinson’s disease, is characterized by slowed movement execution, often accompanied by reduced movement amplitude and the sequence effect, i.e. a progressive reduction of movement amplitude and velocity during movement repetition.^1^ Similar to other motor symptoms in Parkinson’s disease, bradykinesia features primarily affect the upper and lower limbs, are typically asymmetrical and may continue to exhibit side asymmetry throughout the progression of the disease.^2-5^ Bradykinesia feature asymmetry is believed to be a result of the unbalanced involvement of the basal ganglia circuits and their projections to the primary motor cortex (M1).^4-8^ Accordingly, previous neurophysiological studies exploring M1 in patients with Parkinson’s disease have revealed distinct cortical activity patterns between the most affected and less affected hemispheres.^4^

Transcranial magnetic stimulation (TMS) studies have provided evidence of changes in corticospinal and intracortical excitability, as well as M1 plasticity, in Parkinson’s disease.^4,9-17^ Differently from healthy subjects who do not have significant interhemispheric differences in TMS measurements,^18-22^ several studies provided evidence that in Parkinson’s disease, these changes differently affect the two M1s,^4,9-16^ e.g. patients have different short-interval intracortical inhibition (SICI) and cortical silent period (CSP) values, as well as different response to TMS plasticity protocols between the most and less affected M1.^9,15^ Interhemispheric differences in Parkinson’s disease have therefore been considered markers of lateralized cortical pathology,^9,10^ and patients with clinically asymmetric Parkinson’s disease have been considered an evaluable model to study compensatory reorganization within the motor system.^8-10^

In addition to M1 excitability and plasticity changes, some studies have demonstrated a decreased interhemispheric connectivity in Parkinson’s disease.^23-25^ A neurophysiological approach to investigate interhemispheric inhibition (IHI) in humans is through paired-pulse TMS techniques.^26-30^ IHI is believed to be mediated by the transcallosal glutamatergic pathways, which interact with the pyramidal tract neurons at the M1 level through GABAA and GABABergic interneurons, depending on the duration of the interstimulus interval (ISI) between the conditioning stimulus (CS) and the test stimulus (TS).^15,17,22,26,27,30-37^ To date, only a few studies assessed IHI in Parkinson’s disease, with controversial results.^38-41^ Li *et al.*^38^ tested IHI from both the most and less affected M1. They also examined the potential impact of IHI on various TMS measures in Parkinson’s disease. They found that some patients might present a reduced IHI. However, other studies found normal IHI in Parkinson’s disease.^39,40^ Overall, the potential relationship between IHI and other TMS measurements and motor symptom asymmetry in patients remains largely unknown. Furthermore, the effects of dopaminergic therapy on IHI and other measures in Parkinson’s disease have not been largely investigated.

In this study, we aimed to investigate IHI in patients with Parkinson’s disease compared to a control group. We specifically focused on examining the relationship between the asymmetry of IHI and other neurophysiological measures of M1 and the asymmetries of bradykinesia features, objectively assessed by kinematic techniques, on both the most and less affected sides of the body. We hypothesize that IHI relates to some bradykinesia features in Parkinson’s disease, as already demonstrated for other TMS measures of corticospinal/intracortical excitability and plasticity.^4,12,15,16,42^ This would help clarify the role of M1 in bradykinesia pathophysiology.^4^ Additionally, we explored the potential influence of dopaminergic therapy on IHI and other neurophysiological measures, including movement kinematics, as well as their possible relationship in patients.

## Materials and methods

### Participants

We screened 23 patients aged 40–85 years diagnosed with Parkinson’s disease according to the latest diagnostic criteria.^3^ These individuals underwent routine outpatient controls at the Department of Human Neurosciences and IRCCS Neuromed, Sapienza, University of Rome, Italy, between 1 September and 31 December 2022. Since our focus was to investigate interhemispheric connectivity related to bradykinesia asymmetry in Parkinson’s disease, as part of our selection criteria, we excluded five patients with a tremor-dominant phenotype. The rationale for focusing on akinetic-rigid Parkinson’s disease was to comprehensively investigate all major bradykinesia features, including the sequence effect, which might be less evident in individuals with the tremor-dominant phenotype of Parkinson’s disease.^43^ Additionally, the presence of rest tremor in the upper limbs could potentially interfere with electromyographic and kinematic recordings. Thus, we enrolled 18 patients with a clinically asymmetric akinetic-rigid Parkinson’s disease (5 females) and 18 healthy controls (HCs) of comparable age and sex (Table 1). Patients were evaluated with the Movement Disorder Society–sponsored revision of the Unified Parkinson’s Disease Rating Scale (MDS-UPDRS),^44,45^ part III, performed with a standardized examination protocol. To define the most and less affected side in patients, we considered the sum of items 3.3–3.8 for each side (lateralized scores).^9^ These specific items regard the evaluation of rigidity and repetitive movements of the upper and lower limbs.^44,45^ In light of previous evidence indicating the involvement of IHI in mirror movements,^30,38,39^ we also conducted a specific assessment to determine the presence of such movements in the enrolled patients. Notably, none of the patients exhibited mirror movements during finger-tapping. A complete clinical neurological examination was performed in HCs. None of the HCs had a history of neurological or psychiatric disorders, nor were they taking any medications. All participants in the study were right handed, as evaluated by the Handedness Questionnaire.^46^ Notably, none of the patients had changed their dominant hand due to symptom severity. No participants had any contraindications for TMS.^37^ All participants underwent a cognitive evaluation using the Montreal Cognitive Assessment (MoCA),^47^ and none of them had a MoCA score below 26. Clinical assessment in Parkinson’s disease also included the Hoehn and Yahr Scale,^48^ the Hamilton Depression Rating Scale (HAM-D),^49^ the Hamilton Anxiety Rating Scale (HAM-A),^50^ the Frontal Assessment Battery (FAB),^51^ and the Fatigue Severity Scale (FSS).^52^ Levodopa equivalent daily dose (LEDD) was calculated in patients.^53^ All participants provided informed consent prior to participating in the experimental procedures. The study was approved by the local institutional review board and conducted in compliance with international safety guidelines.^37^ The study adhered to the ethical standards outlined by the Declaration of Helsinki.

**Table 1:** Demographic and clinical data of patients with Parkinson’s disease and HCs

	Parkinson’s disease (*n* = 18)	HCs (*n* = 18)	*P*-value
Sex	5 F	10 F	0.09
Age	67.56 ± 7.77	72.96 ± 7.1	0.74
MoCA	26.78 ± 1.26	27.22 ± 2.13	0.06
Disease duration	5.56 ± 4.26		
Motor symptoms onset body side	11 right/7 left		
Most affected body side	11 right/7 left		
MDS-UPDRS part III (OFF/ON condition)	25.85 ± 11.93/18 ± 5.64		
HAM-A	9.78 ± 7.71		
HAM-D	5.67 ± 4.50		
FAB	16.89 ± 1.2		
FSS	25.33 ± 15.86		
LEDD	434.44 ± 181.03		

Age and disease duration are expressed in years. The most affected body side is meant at the time of evaluation. Results are shown as mean values ± 1 standard deviation (SD). *P*-values by parametric and non-parametric comparisons between Parkinson’s disease and healthy controls (HCs). F, females; MoCA, Montreal Cognitive Assessment; MDS-UPDRS part III, Movement Disorder Society–sponsored revision of the Unified Parkinson’s Disease Rating Scale, part III; HAM-A, Hamilton Anxiety Rating Scale; HAM-D, Hamilton Depression Rating Scale; FAB, Frontal Assessment Battery; FSS, Fatigue Severity Scale; LEDD, levodopa equivalent daily dose.

### TMS

Single and paired-pulse TMS were administered to both the right and left M1 using a Magstim 200 stimulator (Magstim Company) connected to a 7 cm figure-of-eight coil. The order of stimulation of the two hemispheres was counterbalanced across participants for all TMS measurements. The coil was held tangentially to the scalp with the handle positioned backward at a 45° angle laterally to the midline inducing a posterior–anterior current in the brain.^30,37,54^ The hotspots of the first dorsal interosseous (FDI) muscles, i.e. the optimal scalp positions for eliciting motor-evoked potentials (MEPs) of maximal amplitudes in the contralateral FDI, as well as resting motor thresholds (RMTs), defined as the lowest TMS intensity able to evoke at least 5 out of 10 MEPs with a >50 μV peak-to-peak amplitude in the relaxed contralateral FDI, were determined according to international guidelines.^55^ The intensity needed to elicit MEPs with an amplitude of ∼1 mV (1mV-MEP) from both the right and left FDI was determined.^11,12,21,22,54^ We collected 15 MEPs using the 1mV-MEP intensity from both FDI muscles. The MEP size was quantified as the peak-to-peak amplitude (mV).

IHI was assessed using paired-pulse TMS connected with two 7 cm figure-of-eight coils.^22,26,29,30,34,36,38,56^ Both hemispheres were tested randomly, with TMS applied to both hemispheres: (i) IHI less-to-most affected M1 and (ii) IHI most-to-less affected M1. Two different ISIs, 10 and 40 ms, were used between the CS and the TS to evaluate short-latency IHI (sIHI) and long-latency IHI (lIHI), respectively.^22,30,31,35^ The intensity of the CS (CS-int) was set at 130% of the RMT, while the intensity of the TS (TS-int) was set at 1mV-MEP intensity (RMT and 1mV-MEP were determined again during the IHI assessment). Fifteen TS and 15 trials for each ISI were randomly collected.

In the same experimental session, in addition to assessing IHI, paired-pulse TMS connected with one coil was used to evaluate the intracortical excitability of the two M1s. In detail, the SICI on both M1s was measured at ISIs of 2 and 4 ms, CS intensity at 80% RMT, and TS intensity at 1mV-MEP (determined again during the SICI assessment).^12,21,57,58^ Fifteen TS and 15 trials for each ISI were randomly collected. IHI and SICI assessments were performed in random order in participants. Both SICI and IHI were quantified as the ratio between conditioned and unconditioned MEPs.^21,22,26,30,34,57,59^

EMG activity was recorded at rest from both FDI muscles. Raw signals were sampled at a rate of 5 kHz using a CED 1401 analogue-to-digital laboratory interface (Cambridge Electronic Design) and amplified and filtered within a bandwidth of 5 Hz–2 kHz using a Digitimer D360 amplifier (Digitimer, Ltd.). The recorded data were stored on a laboratory computer for subsequent offline analysis. The Signal software (Cambridge Electronic Design) was employed for the offline analysis conducted by a researcher (M.D.R.) blinded to the experimental conditions.

### Kinematic recording and analysis

We captured the kinematics of repetitive finger-tapping using a 3D optoelectronic system (SMART motion system, BTS, Milan, Italy). The system consisted of three infrared cameras (sampling rate of 120 Hz). Reflective markers, measuring 5 mm in diameter and of negligible weight, were securely attached to the participant’s hand.^12,16,42,60-64^ Participants were seated comfortably in a chair and instructed to perform repetitive tapping of their index finger on their thumb for 15 s. Three consecutive trials of 15 s each were recorded for each hand, with the order of trials randomized. To prevent fatigue, participants were provided with a rest period of 45–60 s between each trial.^12,16,42,60-64^ The kinematic data were blindly analysed using specialized software (SMART Analyzer, BTS, Milan, Italy). We measured the number of movements and movement rhythm quantified by the coefficient of variation (CV) of the intertap intervals.^12,16,42,60-64^ Linear regression analysis was employed to estimate movement amplitude (degrees), movement velocity (degrees per second), and the decrement in amplitude and velocity (i.e. sequence effect) observed across the 15-s trials.^12,16,42,60-64^

### Experimental design

Patients participated in two experimental sessions: (i) under their usual therapeutic regimen (ON condition) and (ii) after overnight withdrawal (at least 12 h) of their medications (OFF condition).^12,16^ The order of the sessions was randomized, with ≥1 week between the two sessions. HCs participated in only one experimental session. Session duration was around 1.5 h. The examiners who collected the neurophysiological measures were unaware of the patients’ medication status, ensuring blinding during the assessments.

### Statistical analysis

The sample size calculation was performed using the G*Power software.^65^ We set a desired power of 0.80 and an alpha error of 0.05, assuming a 20% change in TMS and kinematic measures between Parkinson’s disease and HCs based on previous studies.^12,16,63^ A sample size of 15 participants was the minimum required to detect a significant difference between groups. Age differences between Parkinson’s disease and HCs were evaluated using the Mann–Whitney U-test, while sex differences were assessed using Fisher’s exact test. Differences in MoCA scores were evaluated with the Mann–Whitney U-test. MDS-UPDRS (part III) scores in patients’ ‘OFF’ and ‘ON’ conditions were compared using the Wilcoxon test.

Group comparisons for RMT, 1mV-MEP and kinematic variables between Parkinson’s disease (OFF medication, most affected hemisphere/side) and HCs (dominant hemisphere/side) were conducted using parametric tests, i.e. two-tailed unpaired *t*-tests. Note that, based on previous studies, we compared the patients’ most affected side with the dominant side in HCs^63,66^; notably, the majority of Parkinson’s disease patients (11 of 18) displayed higher motor impairment on their dominant side (Table 1). Hence, handedness was not considered a significant factor based on previous research demonstrating no significant impact on neurophysiological and kinematic measures.^12,16,22,63^ Group comparisons on IHI and SICI values were evaluated with repeated-measures ANOVAs (rmANOVAs) with the between-group ‘GROUP’ factor (Parkinson’s disease and HCs) and the within-group ‘ISI’ factor (10 and 40 ms for IHI, 2 and 4 ms for SICI).

To test possible hemispheric differences in patients, as well as the effects of medications, we performed additional ANOVAs on RMT and 1mV-MEP using the within-group ‘HEMISPHERE’ (‘most affected’ and ‘less affected’) and ‘SESSION’ (‘OFF’ and ‘ON’) factors. For IHI and SICI analyses, the ‘ISI’ factor was added. For kinematic data, again, we used the within-group ‘SESSION’ (‘OFF’ and ‘ON’) and ‘SIDE’ factors (‘most affected’ and ‘less affected’).

We assessed normal distribution of neurophysiological data with Shapiro–Wilk’s test, and Greenhouse–Geisser corrections were applied whenever we found a violation of sphericity in Mauchly’s tests. Unless otherwise specified, all results are expressed as mean values ± standard error of the mean, and the significance level for all tests was set at *P* < 0.05. Pairwise comparisons were corrected by the Tukey test.

We calculated Spearman’s correlation coefficient to evaluate possible clinical, TMS and kinematic data associations. To this aim, we computed asymmetry indices (AI) [AI = (less affected − most affected)/(less affected + most affected)] of the collected neurophysiological measures (IHI and SICI).^9,10,38,67^ The higher the AI value, the more asymmetric the measure. In the case of IHI-AI, the greater the IHI-AI value, the lower the inhibition less to the most as compared to the inhibition most to the less affected M1. Then, we test whether each neurophysiological AI correlated with clinical scores and kinematic data in patients. Results were corrected for multiple comparisons using the false discovery rate (FDR).^68^ All data were analysed using STATISTICA (TIBCO Software Inc., Palo Alto, CA, USA).

## Results

All participants completed the experimental procedures with no adverse effects. There was no difference in age (*P* = 0.74), sex distribution (*P* = 0.09), or MoCA scores (*P* = 0.06) between Parkinson’s disease patients and HCs (Table 1). As expected, the MDS-UPDRS part III score in Parkinson’s disease was higher in the ‘OFF’ medication session compared to the ‘ON’ session (*P* < 0.01; Table 1). In 11 out of 18 patients, the right side was identified as the most affected in terms of motor symptoms. Importantly, for all patients, the body side with the most pronounced motor symptoms at the time of evaluation corresponded with the side where symptoms initially manifested at disease onset.

### TMS

#### Parkinson’s disease patients’ ‘OFF’ medication versus HCs

**Figure 1:**
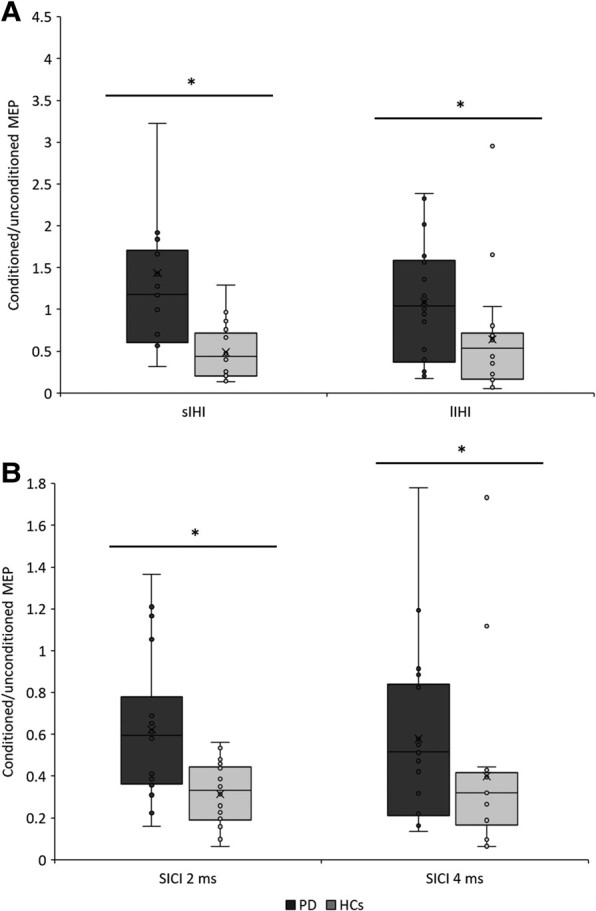
**IHI and SICI in Parkinson’s disease patients and HCs.** (**A**) sIHI with an ISI between the CS and the TS of 10 ms. lIHI with an ISI of 40 ms. The figure shows data collected with the TS delivered on the most affected M1 in patients and on the dominant M1 in HCs. (**B**) SICI was assessed using two ISIs: 2 and 4 ms. The figure shows data collected from the most affected M1 in patients and from the dominant M1 in HCs. SICI and IHI values are expressed as the ratio of conditioned MEPs/unconditioned MEPs. Horizontal lines denote the median values, and ‘×’ denotes the average values. The boxes contain the 25th–75th percentiles of the data set. Asterisks indicate significant *P*-values by *post hoc* comparisons of two rmANOVAs {IHI: ‘GROUP’ [*F*(1,34) = 8.45, *P* = 0.006], ‘ISI’ [*F*(1,34) = 0.58, *P* = 0.45], ‘GROUP’ × ‘ISI’ [*F*(1,34) = 3.99, *P* = 0.06]; SICI: ‘GROUP’ [*F*(1,34) = 5.73, *P* = 0.02], ‘ISI’ [*F*(1,34) = 0.13, *P* = 0.72], and ‘GROUP’ × ‘ISI’ [*F*(1,34) = 1.27, *P* = 0.26]}. CS, conditioning stimulus; HCs, healthy controls; lIHI, long-latency IHI; ISI, interstimulus interval; M1, primary motor cortex; MEPs, motor evoked potentials; rmANOVA, repeated-measures analyses of variance; SICI, short-interval intracortical inhibition; sIHI, short-latency interhemispheric inhibition (IHI); TS, test stimulus.

RMT (*P* = 0.24) and 1mV-MEP values (*P* = 0.07) did not statistically differ between Parkinson’s disease and HCs (Table 2). The rmANOVA on IHI showed a significant effect of the ‘GROUP’ factor [*F*(1,34) = 8.45, *P* = 0.006]. The ‘ISI’ factor and the ‘GROUP’ × ‘ISI’ interaction were not significant [*F*(1,34) = 0.58, *P* = 0.45 and *F*(1,34) = 3.99, *P* = 0.06, respectively]. As shown in Table 2 and Fig. 1A, Parkinson’s disease patients had higher values (indicating less inhibition) of both sIHI and lIHI compared to controls (*P* = 0.04 and *P* = 0.02, respectively). The rmANOVA on SICI showed a significant effect of the ‘GROUP’ factor [*F*(1,34) = 5.73, *P* = 0.02]. This was due to higher SICI values in patients than in controls (Table 2 and Fig. 1B), as shown by *post hoc* comparisons. Again, the ‘ISI’ factor and the ‘GROUP’ × ‘ISI’ interaction were not significant [*F*(1,34) = 0.13, *P* = 0.72 and *F*(1,34) = 1.27, *P* = 0.26, respectively].

**Table 2:** TMS and kinematic variables of finger-tapping movements in patients with Parkinson’s disease and HCs

	Parkinson’s disease				HCs
	OFF condition		ON condition		
	MA	LA	MA	LA	DM
RMT	45.94 ± 10.46	46.72 ± 10.29	43.84 ± 9.79	45.61 ± 7.99	50.28 ± 11.49
1mV-MEP	1.25 ± 0.24	1.23 ± 0.36	1.13 ± 0.40	1.25 ± 0.42	1.04 ± 0.41
sIHI	1.44 ± 1.24	1.46 ± 1.32	1.23 ± 0.97	1.27 ± 1.08	0.48 ± 0.33
lIHI	1.09 ± 0.71	1.13 ± 1.28	1.13 ± 0.85	1.31 ± 1.22	0.64 ± 0.71
SICI 2 ms	0.62 ± 0.35	0.58 ± 0.42	0.73 ± 0.52	0.55 ± 0.30	0.31 ± 0.15
SICI 4 ms	0.56 ± 0,54	0.69 ± 0.67	0.70 ± 0.55	0.59 ± 0.25	0.40 ± 0.41
N. MOV	46.84 ± 14.48	52.80 ± 12.29	48.47 ± 17.05	50.62 ± 16.27	45.8 ± 13.61
CV (rhythm)	0.15 ± 0.08	0.15 ± 0.09	0.13 ± 0.08	0.13 ± 0.06	0.09 ± 0.03
Movement amplitude	44.01 ± 9.62	49.83 ± 8.51	45.0 2 ± 14.42	50.98 ± 11.92	52.69 ± 10.43
Movement velocity	856.11 ± 243.71	895.18 ± 477.45	966 ± 277.81	1103.52 ± 232.54	1106.54 ± 187.41
Amplitude decrement	−0.26 ± 0.21	−0.18 ± 0.23	−0.25 ± 0.32	−0.25 ± 0.34	−0.02 ± 0.21
Velocity decrement	−6.62 ± 4.35	−5.36 ± 4.38	−5.11 ± 4.15	−6.72 ± 7.31	−6.96 ± 4.64

Note that in the case of IHI, the hemisphere refers to the one where the TS was applied. Movement amplitude is expressed in degrees. Movement velocity is expressed in degrees per second. Amplitude decrement is expressed in degree per number of movements. Velocity decrement is expressed in (degrees per second)/number of movements. Results are shown as mean values ± 1 standard deviation (SD). TMS, transcranial magnetic stimulation; HCs, healthy controls; MA, most affected hemisphere/hand; LA, less affected hemisphere/hand; DM, dominant hemisphere/hand; RMT, resting motor threshold, expressed as percentage of the maximal stimulator output (MSO); 1mV-MEP, motor-evoked potentials collected at the stimulation intensity able to elicit MEPs with an amplitude of approximately 1 mV; sIHI, short-latency interhemispheric inhibition, with an ISI between the CS and the TS of 10 ms; lIHI, long-latency IHI, with an ISI of 40 ms; SICI, short-interval intracortical inhibition, assessed using two ISIs: 2 and 4 ms; N. MOV, number of movements; CV, coefficient of variation.

#### Parkinson’s disease patients’ ‘most affected’ versus ‘less affected’ hemisphere (‘OFF’ and ‘ON’ medication)

RMT and 1mV-MEP did not differ between hemispheres nor conditions in Parkinson’s disease [Table 2; RMT: ‘HEMISPHERE’: *F*(1,17) = 1.10, *P* = 0.31; ‘SESSION’: *F*(1,17) = 2.06, *P* = 0.17; ‘HEMISPHERE’ × ‘SESSION’: *F*(1,17) = 0.29, *P* = 0.59; 1mV-MEP: ‘HEMISPHERE’: *F*(1,17)= 0.56, *P* = 0.46; ‘SESSION’: *F*(1,17) = 0.44, *P* = 0.51; ‘HEMISPHERE’ × ‘SESSION’: *F*(1,17) = 1.06, *P* = 0.31].

The rmANOVA on IHI did not reveal any significant factors or interactions [‘SESSION’: *F*(1,17) = 0.04, *P* = 0.84; ‘HEMISPHERE’: *F*(1,17) = 0.93, *P* = 0.76; ‘ISI’: *F*(1,17) = 3.92, *P* = 0.06; ‘SESSION’ × ‘ISI’: *F*(1,17) = 3.68, *P* = 0.72; ‘SESSION’ × ‘HEMISPHERE’: *F*(1,17) = 0.21, *P* = 0.88; ‘HEMISPHERE’ × ‘ISI’: *F*(1,17) = 0.16, *P* = 0.69; ‘SESSION’ ×‘HEMISPHERE’ × ‘ISI’: *F*(1,17) = 0.27, *P* = 0.61; Table 2]. Similarly, the rmANOVA on SICI values did not show any significant factors or interactions [‘SESSION’: *F*(1,17) = 0.09, *P* = 0.76; ‘HEMISPHERE’: *F*(1,17) = 0.61, *P* = 0.44; ‘ISI’: *F*(1,17) = 0.3, *P* = 0.59; ‘SESSION’ × ‘ISI’: *F*(1,17) = 0.15, *P* = 0.7; ‘SESSION’ × ‘HEMISPHERE’: *F*(1,17) = 0.79, *P* = 0.38; ‘HEMISPHERE’ × ‘ISI’: *F*(1,17) = 2.04, *P* = 0.17; ‘SESSION’ × ‘HEMISPHERE’ × ‘ISI’: *F*(1,17) = 0.29, *P* = 0.59; Table 2].

### Finger-tapping kinematics

#### Parkinson’s disease patients’ ‘OFF’ medication versus HCs

The analysis showed a between-group difference in movement amplitude and velocity, with lower values observed in patients compared to controls (*P* = 0.01 and *P* < 0.01, respectively; Table 2). Additionally, there was a difference in movement rhythm, as indicated by higher CV values in Parkinson’s disease (*P* = 0.01), as well as in the amplitude slope (sequence effect; *P* < 0.01), with higher values observed in Parkinson’s disease patients compared to HCs (Table 2).

#### Parkinson’s disease patients’ ‘most affected’ versus ‘less affected’ side (‘OFF’ and ‘ON’ medication)

A significant effect of the ‘SIDE’ factor was found for movement amplitude [*F*(1,17) = 8.92, *P* < 0.01], with lower values on the most affected compared to the less affected side (Table 2), with no effect of the ‘SESSION’ factor [*F*(1,17) = 0.23, *P* = 0.64] or a ‘SESSION’ × ‘SIDE’ interaction [*F*(1,17) = 0.01, *P* = 0.97]. There was also a significant effect of the ‘SESSION’ for movement velocity [‘SESSION’: *F*(1,17) = 5.5883, *P* = 0.03], with lower velocity values observed during the OFF compared to the ON session (Table 2). Although velocity values were lower on the most affected side, no significant effect of ‘SIDE’ [*F*(1,17) = 1.67, *P* = 0.21] or the ‘SESSION’ × ‘SIDE’ interaction [*F*(1,17) = 0.85, *P* = 0.37] was found. More detailed analysis data are in Supplementary Table 1.

### Correlation analysis

**Figure 2:**
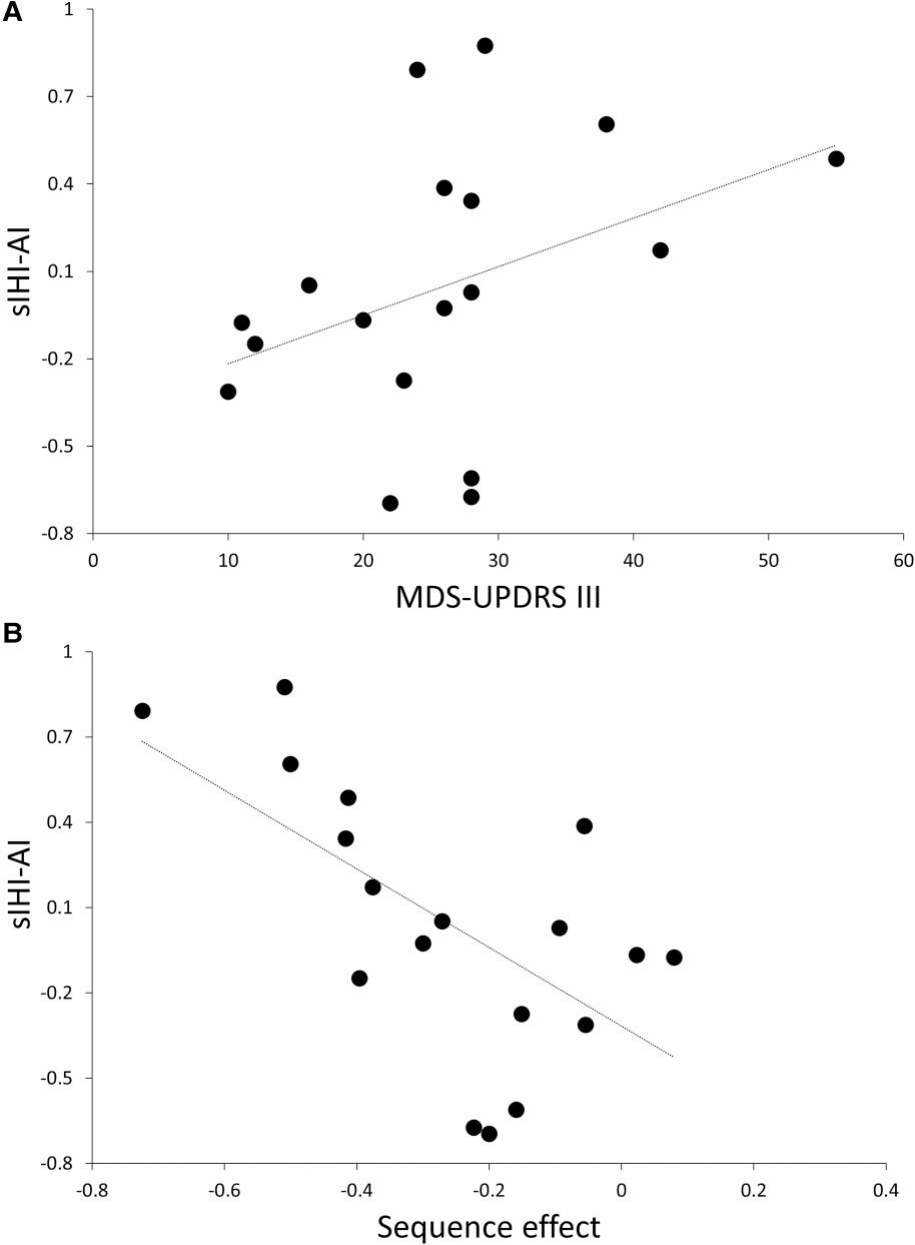
**Correlations between neurophysiological, clinical and kinematic data in Parkinson's disease**. (**A**) Motor section (part III) of the MDS-UPDRS-III scores (*x*-axis) and sIHI-AI (*y*-axis). sIHI-AI was calculated as follows: AI = (sIHI from the less to the most affected hemisphere − sIHI from the most to the less affected hemisphere)/(sIHI from the less to the most affected hemisphere + sIHI from the most to the less affected hemisphere); *r* = 0.5; *P* = 0.034. (**B**) Sequence effect (finger tapping movement—most affected hand; *x*-axis), expressed in degree/number of movements, and sIHI-AI (y-axis); *r* = −0.61; *P* < 0.01. MDS-UPDRS, Movement Disorder Society–sponsored revision of the Unified Parkinson's Disease Rating Scale; sIHI-AI, short-latency interhemispheric inhibition-asymmetry index.

**Figure 3:**
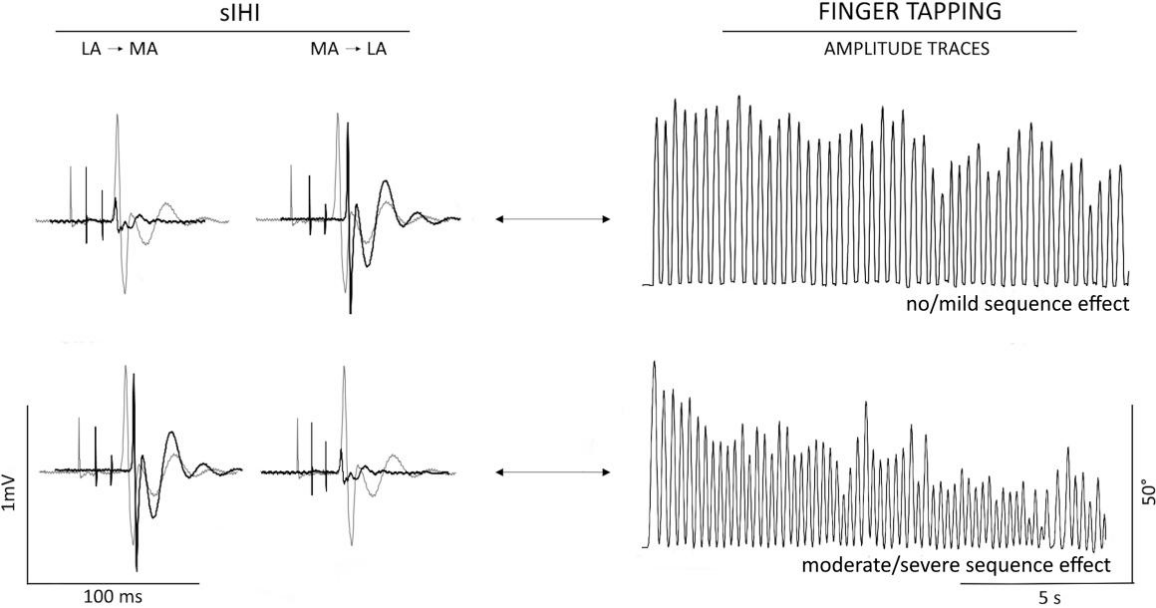
**Schematic illustration of the main study finding in Parkinson’s disease.** LA, less affected hemisphere; MA, most affected hemisphere; sIHI, short-latency interhemispheric inhibition. Lighter motor evoked potentials (MEPs) indicate unconditioned MEPs. Darker MEPs indicate conditioned MEPs during the sIHI assessment (with an ISI of 10 ms between the conditioning and the TS). Note that patients with a preserved sIHI from the LA to the MA hemisphere showed no/mild amplitude decrement (no/mild sequence effect) at the kinematic recordings of finger-tapping movements with the MA hand. Conversely, Parkinson’s disease patients with reduced sIHI from the LA to the MA hemisphere had a more pronounced sequence effect during the finger-tapping performed with the MA hand.

We observed a positive correlation between the sIHI-AI and the MDS-UPDRS part III score in patients ‘OFF’ condition (*r* = 0.5, *P* = 0.034; Fig. 2A). The greater the IHI imbalance towards the less affected hemisphere (i.e. less inhibition from the less to the most affected M1 than that measured from the most to the less affected MI), the higher the MDS-UPDRS-III score. However, the present correlation did not survive to the FDR correction (FDR-adjusted *P* < 0.01). Again, we found a significant negative correlation between the sIHI-AI and the sequence effect of the most affected side in Parkinson’s disease patients tested during the ‘OFF’ condition (*r* = −0.61, *P* < 0.01; Fig. 2B). This indicates that the greater the imbalance of sIHI abnormality towards the less affected hemisphere, the more pronounced the sequence effect of the most affected hand (Fig. 3). The present correlation was not observed for lIHI-AI values or during the ‘ON’ session. No other correlations were found between clinical, TMS and kinematic data (all *P* > 0.05).

## Discussion

In this study, we investigated changes of IHI and other TMS measures of M1 in Parkinson’s disease patients, focusing on interside differences. Again, we examined the relationship between IHI and bradykinesia feature asymmetry. Finally, we investigated the effects of dopaminergic therapy on these relationships. Our findings revealed reduced sIHI and lIHI in Parkinson’s disease patients compared to controls, with no significant hemispheric difference. When evaluating repetitive finger movements using kinematic analysis, we found that the imbalance of sIHI (less inhibition from the less to the most affected M1 than that measured from the most to the less affected M1) was related to the sequence effect of the most affected hand. Also, we observed a trend to a positive correlation between the Parkinson’s disease group’s sIHI imbalance and MDS-UPDRS-III scores. These results may suggest that IHI imbalance may contribute to bradykinesia features in Parkinson’s disease patients. While dopaminergic therapy overall improved bradykinesia, it did not restore the impaired IHI nor the sequence effect as kinematically evaluated. However, dopaminergic therapy influenced the relationship between sIHI imbalance and bradykinesia in Parkinson’s disease patients.

Given the similarities in demographic characteristics between the Parkinson’s disease and HCs groups, we exclude these factors as potential confounding. While the diagnosis of Parkinson’s disease was based on clinical criteria and not all patients underwent a DaTscan examination, it is important to note that all patients were consistently monitored in our outpatient clinic over an extended period, minimizing the misdiagnosis bias.^3,69^ To ensure the precision of our evaluations, we implemented additional measures. In order to assess bradykinesia, we utilized finger-tapping movements, the most valuable task in clinical practice.^1,4,12,44,45,70^ Notably, we tested patients under their usual dopaminergic therapy and after they discontinued the therapies 12 h before the experimental evaluation. Finally, although the examiners who collected the neurophysiological measures were not blinded to the participants’ clinical status (Parkinson’s disease patients were tested twice while HCs underwent only one experimental session), they were unaware of the patients’ medication status. Most importantly, the researcher who performed TMS and kinematic analyses was blinded to the participants’ diagnosis (Parkinson’s disease versus HCs) and to the experimental condition (‘ON’ versus ‘OFF’ condition).

One novel finding of this study was the presence of altered IHI in Parkinson’s disease. Also, our study adds to the existing literature by specifically highlighting the asymmetry and variability of various excitability measures of M1 between the two hemispheres.^4,6,9,10,15^ Only a few studies, to date, have tested possible alterations of the connectivity between the two M1, as assessed by IHI,^38-40^ and the provided results are controversial. Most of these studies investigated the relation between IHI and mirror movements in patients, based on the hypothesis that mirror movements are due to motor overflow between hemispheres possibly reflecting an altered IHI.^38,39^ For example, it has been observed a reduced IHI in patients with mirror movements compared to those without mirror movements. However, when considering the entire group of Parkinson’s disease patients, an overall increase in IHI was found compared to the HCs.^38^ Also, the authors did not specifically analyse the correlation between IHI and bradykinesia asymmetry in patients.^38^ Moreover, it is worth noting that Li *et al.* tested patients in the ‘OFF’ dopaminergic condition only. Again, Zittel *et al.*^40^ described a trend for reduced IHI in Parkinson’s disease *de novo* patients tested ON medication as compared with a control group, while other authors found normal IHI in Parkinson’s disease.^39^ Differences in terms of patients’ population, e.g. patients with and without mirror movements, as well as in the experimental conditions between these previous studies and ours may explain such a variability of the results. Our data of a reduced IHI in Parkinson’s disease can be discussed also in regard to other neurophysiological studies, which assessed the interhemispheric interactions by means of the ipsilateral silent period (iSP).^71,72^ As for the IHI, the iSP is thought to be the result of an interhemispheric inhibitory transfer mediated by callosal fibers.^15,22,41,71-73^ iSP duration was longer when the more affected hemisphere was stimulated compared to the less affected hemisphere in patients.^71,72^ The weaker iSP recorded from the less affected side was interpreted as a reduced ability of the worse M1 to adequately inhibit the opposite M1. Again, levodopa restored this interhemispheric iSP difference.^71^ Finally, the altered inhibition between the two hemispheres we found in Parkinson’s disease patients agrees with previous neuroimaging data.^23-25^ In this regard, analytic approaches of resting-state data examined the functional relationship between remote brain regions and revealed a decreased interhemispheric M1 connectivity in Parkinson’s disease.^23-25^ Accordingly, altered functional brain connectivity has recently been considered a Parkinson’s disease hallmark.^74^

The pathophysiological role of the reduced IHI can be further discussed in relation to the correlation analysis. Remarkably, we observed a trend to a correlation between the sIHI-AI and the patients’ global MDS-UPDRS part III scores. The greater the imbalance of sIHI abnormality towards the less affected hemisphere (i.e. less inhibition from the less to the most affected M1 than that measured from the most to the less affected M1), the higher the MDS-UPDRS-III score. This correlation was further elucidated when examining bradykinesia using the kinematic techniques. Specifically, the greater the imbalance of sIHI abnormality towards the less affected hemisphere, the more pronounced the sequence effect of the most affected side. Importantly, using objective measurements, this study is the first to investigate the relationship between IHI and altered movement asymmetry in Parkinson’s disease. The findings may suggest that an imbalance in interhemispheric connections possibly contributes to some bradykinesia features in Parkinson’s disease. These results support the hypothesis that functional changes predominantly occurring on the less affected side in Parkinson’s disease may play a role in preventing the progression of motor symptoms.^9,10^ In light with this hypothesis, we may speculate that the sIHI from the less to the most affected hemisphere in Parkinson’s disease serves as a compensatory mechanism for motor dysfunction. Indeed, when the inhibition decreases and the mechanism of IHI is compromised, the sequence effect becomes more apparent. However, our study did not observe a significant difference in sIHI between the most and less affected sides, which would have provided further support for the hypothesis of IHI acting as a compensatory mechanism for motor impairment.^9,10^ Therefore, this aspect requires further clarification.

The present study provides insights into the pathophysiological interpretation of the sequence effect, one of the key but underinvestigated features of bradykinesia in Parkinson’s disease.^4^ Previous reports demonstrated a relationship between the sequence effect and M1 plasticity abnormalities.^12^ However, it is important to acknowledge that the dysfunction of other brain areas within a broader neural network may also contribute to the pathophysiology of the sequence effect.^4,70^ Our study is the first to demonstrate a relationship between the sequence effect and interhemispheric inhibitory connections in patients. Notably, this relationship was specific to sIHI values and not observed for lIHI values. These findings suggest that different interneuronal circuits may mediate the two neurophysiological measures,^22,30,31,33,75^ and that only sIHI is involved in fine finger movements.^22,30^ In this regard, a recent study performed on a patient with Parkinson’s disease and corpus callosum agenesis found that sIHI was absent, suggesting that callosal pathways are necessary for shorter-latency interhemispheric transfer, while lIHI was preserved.^41^ Finally, in our study, we found that dopaminergic therapy did not ameliorate IHI, neither did the sequence effect.^4,12^ However, we here found that levodopa modified the relationship between sIHI-AI and sequence effect, which was no longer observed when testing patients in their ON condition. This may suggest an influence of the central dopaminergic tone in regulating the interhemispheric connections.

Regarding the potential limitations of our study, it should be noted that we exclusively included patients with the akinetic-rigid form of Parkinson's disease, and therefore, further investigations are warranted to determine the generalizability of our findings to patients with a tremor-dominant phenotype. Additionally, our study focused on patients in the early-moderate stages of Parkinson’s disease, and future research involving patients at different disease stages and longitudinal studies will provide insights into potential changes in the relationship between IHI and bradykinesia features as the disease progresses. Again, our study exclusively involved right-handed participants.^46^ While prior research has shown that handedness is not a significant factor affecting neurophysiological and kinematic measures,^12,16,22,63^ it is acknowledged that handedness can impact manual dexterity^76-78^ and the activation of motor areas during both right and left finger movements.^79-81^ Therefore, further investigations involving individuals with diverse hemispheric dominance are warranted. To ensure a reasonable duration for the experimental procedures, our TMS assessment only included selected neurophysiological measurements. We solely examined IHI and did not assess other TMS measures, such as intracortical facilitation and M1 plasticity, which are known to be specifically influenced by the activation of transcallosal pathways.^22^ Lastly, we did not investigate possible correlations between the time of l-dopa withdrawal (e.g. in hours) and the IHI alterations. As a further comment concerning TMS measures, we did not observe a correlation between altered sIHI and reduced SICI, which is also mediated by postsynaptic GABAA mechanisms.^28,29^ To date, only one previous study has investigated the possible interactions between IHI and SICI in Parkinson’s disease,^38^ testing how IHI modulates SICI in patients without mirror movements. Li *et al.*, however, did not test the basal relationships between these two parameters, so the present topic requires further investigation.

## Conclusion

Our study has provided evidence of reduced IHI in Parkinson’s disease. We have also demonstrated the possible relationship between interhemispheric disinhibition, from the less to the most affected hemisphere, and the severity of the sequence effect in Parkinson’s disease. Overall, if confirmed by future studies, our result could have implications also from a practical and therapeutic standpoint. Specifically, our findings suggest that the imbalance in IHI could be regarded as a neurophysiological marker indicating the severity of bradykinesia in Parkinson’s disease. Furthermore, our data have the potential to contribute to enhancing existing neuromodulation approaches. One hypothesis is that by modulating the IHI balance between the two M1 using non-invasive brain stimulation techniques, a beneficial impact on the sequence effect in Parkinson’s disease patients could be achieved.

## Supplementary Material

fcae020_Supplementary_Data

## Data Availability

The data supporting this study’s findings are available upon request from the corresponding author.
