# Supplementary material for: Interhemispheric imbalance and bradykinesia features in Parkinson’s disease
Source: Brain Commun. 2024 Jan 29;6(1):fcae020. doi: 10.1093/braincomms/fcae020 (PMC10873583; doi:10.1093/braincomms/fcae020)
Supplement: fcae020_Supplementary_Data [file fcae020_supplementary_data.docx]

| **Kinematic parameters** | SESSION | SIDE | SIDE x SESSION |
| --- | --- | --- | --- |
| **N. mov** | F_1,17_=0.12, p=0.72 | F_1, 17_=1.8, p=0.19 | F_1, 17_=1.4, p=0.25 |
| **CV (Rhythm)** | F_1, 17_=1.8, p=0.19 | F_1,17_=0.02, p=0.88 | F_1,17_=0.52, p=0.48 |
| **Movement amplitude** | F_1,17_=0.23, p=0.64 | F_1, 17_=8.92, p<0.01 | F_1, 17_=0.01, p=0.97 |
| **Movement Velocity** | F_1,17_=5.58, p=0.03 | F_1,17_=1.67, p=0.21 | F_1,17_=0.85, p=0.37 |
| **Amplitude decrement** | F_1,17_=0.3, p=0.59 | F_1,17_=0.26, p=0.61 | F_1, 17_=0.76, p=0.39 |
| **Velocity decrement** | F_1,17_=0.01, p=0.94 | F_1,17_=0.01, p=0.91 | F_1,17_=2.97, p=0.1 |

**Supplementary Table 1.** Detailed kinematic results. Data from the dominant and non-dominant side in Parkinson’s disease (PD) patients and healthy controls (HCs) were analyzed with a repeated-measures analysis of variance (rmANOVA) using the factors ‘SIDE (two levels: most vs less affected) and ‘SESSION’ (two levels: ‘OFF’ and ‘ON’ medication) N. mov: number of movements. CV: coefficient of variation.
